# Serotonin Transporter Genotype (*5-HTTLPR*) Predicts Utilitarian Moral Judgments

**DOI:** 10.1371/journal.pone.0025148

**Published:** 2011-10-05

**Authors:** Abigail A. Marsh, Samantha L. Crowe, Henry H. Yu, Elena K. Gorodetsky, David Goldman, R. J. R. Blair

**Affiliations:** 1 Department of Psychology, Georgetown University, Washington, District of Columbia, United States of America; 2 Mood & Anxiety Program, National Institute of Mental Health, Bethesda, Maryland, United States of America; 3 Section of Human Neurogenetics, National Institute on Alcohol Abuse & Alcoholism, Bethesda, Maryland, United States of America; Wayne State University, United States of America

## Abstract

**Background:**

The psychological and neurobiological processes underlying moral judgment have been the focus of extensive recent research. Here we show that serotonin transporter (*5-HTTLPR*) genotype predicts responses to moral dilemmas featuring foreseen harm to an innocent.

**Methodology/Principal Findings:**

Participants in this study judged the acceptability of actions that would unintentionally or intentionally harm an innocent victim in order to save others' lives. An analysis of variance revealed a genotype × scenario interaction, *F*(2, 63) = 4.52, *p* = .02. Results showed that, relative to long allele homozygotes (LL), carriers of the short (S) allele showed particular reluctance to endorse utilitarian actions resulting in foreseen harm to an innocent individual. LL genotype participants rated perpetrating unintentional harm as more acceptable (*M* = 4.98, *SEM* = 0.20) than did SL genotype participants (*M* = 4.65, *SEM* = 0.20) or SS genotype participants (*M* = 4.29, *SEM* = 0.30). No group differences in moral judgments were observed in response to scenarios featuring intentional harm.

**Conclusions/Significance:**

The results indicate that inherited variants in a genetic polymorphism that influences serotonin neurotransmission influence utilitarian moral judgments as well. This finding is interpreted in light of evidence that the S allele is associated with elevated emotional responsiveness.

## Introduction

Judging moral dilemmas typically requires weighing the relative merits of two mutually exclusive outcomes, such as choosing to save many lives even if doing so requires the death of an innocent person. In the face of actual moral dilemmas, individuals' judgments about the optimal course of action to be taken can vary widely [1]. For example, medical researchers investigating new drugs may disagree about the moral acceptability of enrolling patients in the placebo arm of clinical trials when it can be foreseen that some of these patients may suffer or die prematurely as a result [2]. The present study assesses a possible source of variation among individuals who are judging moral dilemmas. We assessed whether genetic variants associated with serotonergic function predict responses to moral dilemmas featuring foreseen and intentional harm.

As is true for actual moral dilemmas, judging moral dilemmas in the laboratory frequently requires weighing alternate outcomes, such as choosing to save many lives by allowing an innocent victim to die. Saving more lives is the more utilitarian option, but the prospect of causing harm to an innocent individual may generate a strong emotional response [3]. Nonetheless, respondents may endorse harming an individual for utilitarian gains when the harm is impersonal rather than personal, or unintentional rather than intentional [4], [5].

Recent research demonstrates that participants' willingness to endorse utilitarian actions that require personally harming an innocent victim can be affected by variables that influence brain functioning, such as lesions of the ventromedial prefrontal cortex and pharmacological challenges [6], [7]. For example, respondents who receive a selective serotonin reuptake inhibitor (citalopram) are less likely to endorse utilitarian outcomes that result in harm to an innocent victim [7]. This may be because serotonin enhances the aversive emotional response to causing others harm, perhaps through its influence on brain structures like the amygdala, insula, and ventromedial prefrontal cortex, which are implicated in moral judgments and behavior [6], [8].

Endogenous serotonin neurotransmission is influenced by a functional 5′ promoter polymorphism of the serotonin transporter (5-HTT) in the human serotonin transporter gene *SLC6A4*, called *5-HTTLPR* [9]. Relative to carriers of the long (L) form of the polymorphism, carriers of the short (S) form show reduced transcription, expression and function of 5-HTT, which influences the reuptake of serotonin from the synaptic cleft [10]. S-carriers are also more emotionally reactive to aversive stimuli than are L-carriers [11]. This difference may reflect S-carriers' increased activation in subcortical structures like the amygdala that are associated with negative affect and/or reduced prefrontal modulation of these structures by the prefrontal cortex [11].

Intentional and foreseen harm are distinguished by the *principle of double effect* [12]. This principle stipulates that foreseen harm that is a side effect (or “double effect”) of bringing about a good result may be permissible. By contrast, intentionally causing harm as a means of bringing about the good end would not be permissible. For example, scenarios in which one moves a switch to divert a train onto a track away from five bystanders, even though it can be foreseen that another person standing on the track will be killed, are usually judged to be permissible. But scenarios in which one moves a switch to drop a person in front of a train, deliberately killing him but saving five people further down the track, are usually judged to be impermissible. We hypothesized that *5-HTTLPR* genotype would interact with intentionality in respondents who generated moral judgments. Whereas we predicted that all participants would eschew intentionally harming an innocent for utilitarian gains, we predicted that participants' judgments of foreseen but unintentional harm would diverge as a function of genotype. Specifically, we predicted that LL homozygotes would adhere to the principle of double effect and preferentially select the utilitarian option to save more lives despite unintentional harm to an innocent victim, whereas S-allele carriers would be less likely to endorse even unintentional harm. Results of behavioral testing confirmed this hypothesis.

## Results

We examined participants' moral judgments using analysis of variance (ANOVA) with genotype as a between-subjects factor and scenario type as a within-subjects factor. When significant main effects or interactions were found, we conducted post hoc *t* tests. Significant differences were set at *p*<.05 (two-tailed). We first conducted a 2 (intentionality)×3 (genotype) ANOVA assessing participants' responses regarding moral acceptability.

### Moral judgments

**Figure 1:**
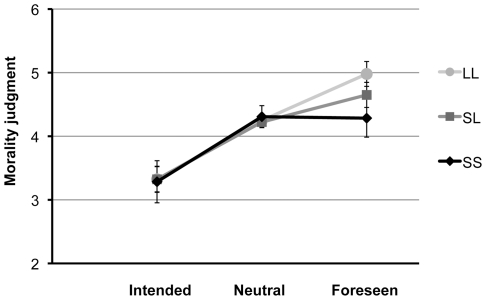
Mean judgments of the moral acceptability of intended, neutral, and foreseen harm by 5-HTTLPR genotype. Error bars denote standard error. Acceptability judgments of foreseen harm scenarios varied linearly with genotype.

In line with predictions, a significant 2 (foreseen, intentional)×3 (SS, SL, and LL genotype) interaction effect emerged, *F*(2, 63) = 4.52, *p* = .02. Tolerance of foreseen harm to a victim varied linearly with genotype (Figure 1). Homozygous L-carriers judged saving five people and causing the foreseen death of an innocent person to be more acceptable than did homozygous S-carriers, *t*(33) = 2.03, *p* = .05. Foreseen harm to an innocent victim was judged to be more acceptable than morally neutral actions by homozyogous L-carriers, *t*(21) = 4.24, *p*<.001, and heterozygotes, *t*(29) = 2.14, *p* = .04. By contrast, homozygous S-carriers judged dilemmas in which harm was foreseen to be no better than morally neutral dilemmas, *t*(12) = −0.07, *p* = .94. Finally, the difference in homozygous L-carriers' responses to scenarios featuring foreseen harm over intentional harm was greater than for homozygous S-carriers, *t*(33) = 2.34, *p* = .03, suggesting LL homozygotes saw a greater moral distinction between the two types of scenarios. As predicted, participants consistently rejected the perpetration of intentional harm to a victim; no group differences were observed in response to these scenarios (all *p*>.90). No group differences were observed in response to neutral scenarios (all *p*>.60) or other comparison dilemmas (all *p*>.10).

### Response times

It has previously been observed that heightened response conflict during moral decision making is associated with longer response times [4], [5]. We thus conducted a 2 (intentionality)×3 (genotype) ANOVA assessing participants' median response times across all response options when they judged foreseen and intentional harm scenarios. A marginally significant main effect of intentionality, *F*(1, 62) = 3.37, *p* = .07, reflected longer response times to foreseen harm scenarios (*M* = 6836 milliseconds, *SD* = 2246) than intentional harm scenarios (*M* = 6540 milliseconds, *SD* = 2612). No main effect of group or interaction was observed (*p*>.10).

More important for our specific hypotheses, we also analyzed variation in response times when participants made different responses. In other words, how did participants' response times vary as a function of how acceptable they judged a course of action to be? To conduct this analysis, we calculated for each participant the correlation between his or her mean response times and the numeric response (1–7) he or she provided for both foreseen and intentional harm scenarios. Thus, a positive correlation indicated that the participant responded more slowly when judging actions to be more acceptable, and a negative correlation indicated that participants responded more slowly when judging the action to be less acceptable. We performed a Fisher transformation on these coefficients to normalize their distribution and compared the resulting coefficients across groups for intentional and foreseen harm using a 2 (intentionality)×3 (genotype) ANOVA. An intentionality × genotype interaction emerged, *F*(2, 58) = 3.42, *p* = .04. In accordance with findings for moral judgments, group differences in response times emerged only when participants judged foreseen harm. Examination of the means indicated that only the responses of homozygous L-carriers varied as a function of response. These participants showed slower response latencies the less acceptable they judged foreseen harm to be (*M* = −0.74, *SD* = 0.69). No relationship between response selection and response times was observed for S-carriers (all *M*<0.15). No main effect of group was found. These patterns suggest that LL homozygotes experienced increased response conflict, and hence delayed response times, when their responses contradicted the principle of double effect.

## Discussion

Accumulating research suggests that serotonergic activity plays an important role in moral reasoning and related social behaviors [7], [13]. *SLC6A4* is a gene that regulates serotonergic activity and has been described as the most investigated genetic variant in the fields of human psychology and neuroscience [14]. However, the association between *5-HTTLPR* genotype and individual differences in moral judgments has not previously been determined. Our results showed that participants agreed that intentionally causing the death of one innocent victim was not a morally acceptable means to a utilitarian end. By contrast, participants' judgments diverged according to genotype when judging foreseen harm. Here, homozygotic carriers of the S allele, which is associated with heightened emotional reactivity and reduced prefrontal regulation of emotion, were less likely than L-carriers to endorse saving many lives if one person would be unintentionally harmed as a result. LL genotype participants judged foreseen harm to be more acceptable, and they responded more slowly when judging foreseen harm to be unacceptable, suggesting increased response conflict in these trials. These findings support our hypothesis that LL genotype participants' moral judgments are more strongly modulated by assessments of intentionality.

These results may aid in understanding why people disagree about the acceptability of causing foreseen harm to meet utilitarian goals. The results of the present study suggest that judgments in response to this kind of moral dilemma may be influenced by inherited variants in a genetic polymorphism that influences serotonin neurotransmission and patterns of responding to socio-emotional stimuli. These findings thus extend previous research in two domains. First, they advance our understanding of how variations of the *5-HTTLPR* influence social cognition. Second, they indicate that a genetic “manipulation” consistently associated with increased emotional responsiveness (the S allele) results in significantly greater reluctance to cause harm to another individual even though others will be helped, and even though harming the innocent is an unintentional aspect of helping. This helps to extend our understanding of the mechanisms underlying moral judgments.

Serotonin function has long been associated with variations in personality and in patterns of affective responding [14]. *5-HTTLPR* S-carriers have been characterized as high in negative affectivity [15], which is defined as a bias toward negatively valenced information and sensitivity to perceived threat [16]. The results of neuroimaging studies suggest that this pattern of responding results from enhanced reactivity of the amygdala to negatively valenced stimuli and/or reduced modulation of this activity by the prefrontal cortex [11]. In the present paradigm, we speculate that these patterns of neural responding, and consequent increased emotional responsiveness (in this case to the plight of the innocent victim), are reflected in S-carriers' reluctance to condone even unintentional harm to an innocent victim despite the possibility of utilitarian gains.

To draw stronger conclusions about the mechanism by which genotype affects moral judgments, it would be optimal to specifically assess correlates of affective responding during moral judgments in S and L-carriers, for example, via psychophysiological or self-report measures of affective responding. Such paradigms could provide support for the notion that the prospect of harming an innocent victim generates an aversive emotional response [3], one that may be enhanced in S-carriers. Neuroimaging studies of the amygdala and prefrontal cortex in S and L-carriers could also test the hypothesis that harming innocents will generate increased amygdala responses in S-carriers relative to LL homozygotes—particularly in response to unintentional but foreseen harm scenarios.

It should be noted that the patterns of response times we observed suggested that LL homozygotes experienced increased response conflict when their responses contradicted the doctrine of double effect, whereas S-carriers did not show consistent differences in response time across conditions. Previous research has demonstrated that when participants judge harming an innocent victim to be acceptable their response latencies are usually slower than when they judge these items to be unacceptable. It has been argued that the emotional response elicited by the prospect of harming an innocent renders the “unacceptable” response the prepotent or dominant response, which then must be overcome when an item is ultimately judged to be acceptable [4]. In the present study, LL homozygotes were significantly more likely than S-carriers to judge actions that resulted in unintentional but foreseen harm to an innocent victim to be acceptable. In other words, judging these actions to be acceptable appeared to be LL homozygotes' dominant response. In addition, LL homozygotes' response latencies were slower the less acceptable they judged foreseen harm to be. This suggests that LL homozygotes responded significantly more slowly the more their responses diverged from their dominant response. This result is consistent with suggestions that response latencies increase under conditions of cognitive conflict [17]. The source of this cognitive conflict cannot be determined definitively from the present paradigm, but it may reflect conflict resulting from the integration of affective responses to harm with information about intentionality.

Recent findings resulting from a paradigm similar to that used in this paper suggest that manipulating the serotonin system influences moral judgments in response to dilemmas featuring personal harm [7]. A strength of the present investigation is that it demonstrates that serotonergic function affects moral reasoning using a distinct set of moral scenarios than those that have been used in several previous studies assessing neuropsychological correlates of moral reasoning [5], [6]. The present findings thus support the generalizability of these prior findings. It should also be noted, however, that it is difficult to directly compare studies in which available serotonin is acutely manipulated with those in which serotonin transporter genotype varies because the mechanism by which *5-HTTLPR* genotype affects serotonergic function is not yet well understood [18]. The polymorphism's most important role may be in modulating responsiveness to stress during development [14]. A precise molecular account of the role of *5-HTTLPR* genotype in moral judgments therefore awaits further characterization. Combining genetic association techniques with acute serotonin manipulation may further elucidate the role of serotonin in moral reasoning, given the known interaction between *5-HTTLPR* genotype and available serotonin levels in emotional responding [17]. However, the current results extend prior observations that moral reasoning can be influenced by pharmaceutical challenges and damage to the brain, finding that individual differences in moral reasoning can also be influenced by inherited genetic variants.

## Materials and Methods

### Participants

Sixty-five healthy volunteers (27 males, 38 females, *M* age = 26.1 years, *SD* = 6.7) took part in this investigation. Participants were recruited via fliers placed in the community that invited them to take part in mental health research. Participants included twenty-two LL genotype subjects, thirty SL genotype subjects, and thirteen SS genotype subjects. Subjects underwent screening at the National Institutes of Health via a standardized psychiatric interview using DSM-IV criteria, a medical history and physical exam performed by a clinician, and blood and urine screening tests. No participants exhibiting current or past major affective disorder, anxiety disorder, psychotic disorder, substance dependence, anorexia nervosa or bulimia were included. Participants were free of psychotropic medications at the time of screening. Urine toxicity screens excluded participants in whom recent drug use was indicated. The matrix reasoning and verbal subtests of the Wechsler Abbreviated Scale of Intelligence were administered to obtain estimated IQ scores and participants with scores <80 were excluded. No IQ differences across groups were observed (all *p*>.20). All participants gave informed written consent and were paid for their participation.

### Ethics Statement

This research was approved by the Combined Neuroscience Institutional Review Board at the National Institute of Mental Health, and all participants' written informed consent was obtained prior to the study's commencement.

### Genotype analysis

DNA for each subject was prepared from saliva samples using Oragene•DNA kits (DNA Genotek, Ottawa, Ontario, Canada). *SLC6A4* gene promoter (*5-HTTLPR*) polymorphism was amplified from 10 ng genomic DNA in a 20 µl reaction: 1× Optimized Buffer A, 1× PCR enhancer, and 0.25 µM each primer (Forward FAM-ATCGCTCCTGCATCCCCCATTAT and Reverse GAGGTGCAGGGGGATGCTGGAA), 0.125 µM dNTP, 10 ng DNA, 1.25 u Platinum Taq polymerase (all Invitrogen Corp). The PCR conditions were 95°C (5 min), 40 cycles of 94°C (30 sec), 52°C (30 sec), 68°C (1 min), and a final elongation, 68°C (10 min). S (103 bp) and L (146 bp) genotypes were discriminated directly from the PCR reaction products. Samples were mixed with deionized formamide and GeneScanTM-500 ROX Size Standard (Applied Biosystems), and genotypes resolved on a 3730 DNA Analyzer (Applied Biosystems). Genotyping accuracy was determined empirically by duplicate genotyping of 25% of the samples selected randomly. The error rate was <.005, and the completion rate was >.95.

### Procedure

Participants made judgments on a set of 150 moral dilemmas, consisting of five versions of 30 distinct scenarios (Table S1). The five versions included two groups of moral scenarios that varied in the intentionality of the harm caused to the victim by the proposed action: intentional harm (*N* = 30) and foreseen harm (*N* = 30). In the versions featuring unintentional harm, saving five lives would mean the foreseen but unintentional death of an innocent victim, for example: pushing a tree into a boulder's path to divert the boulder away from five people but onto an innocent victim. In the intentional harm scenarios, killing the innocent victim was an intentional element of saving five others, for example: pushing down a tree in which an innocent victim is sitting so the victim falls into the path of a boulder that is rolling toward five people. Both intentional harm and foreseen harm versions of each scenario featured an identical action to be taken by the participant (for example, pushing down a tree, throwing a switch, or turning a wheel). There were also three non-moral control scenarios: neutral (e.g., pushing a tree so that it will knock a boulder away, enabling access to a path); no gain (e.g., pushing a tree to knock a person into a boulder's path without any gain to others); and no cost (e.g., pushing a tree to knock a boulder so that it will not hit five people without any cost to anyone else).

Initial validation of these stimuli was accomplished by presenting the stimuli to an independent group of thirteen subjects and assessing ratings of moral acceptability. Actions described in the foreseen harm scenarios were rated as significantly more acceptable than the actions described in intended harm scenarios (means were 4.9 and 4.4 on a 7-point scale); *t*(12) = 4.36, *p*<.001).

In the present study, the text of each scenario was presented on three consecutive screens. The first two screens described the hypothetical scenario and the third presented a question about the action to be taken (“How acceptable would it be to….”). Participants pressed numeric keys 1 (least acceptable) through 7 (most acceptable) to answer each question. The task was self-paced.

## Supporting Information

Table S1 **Example scenarios.** Three examples are provided for each of the five types of moral dilemmas viewed by study participants.
(DOCX)
