# Supplementary material for: Serotonin Transporter Genotype (5-HTTLPR) Predicts Utilitarian Moral Judgments
Source: PLoS One. 2011 Oct 5;6(10):e25148. doi: 10.1371/journal.pone.0025148 (PMC3187753; doi:10.1371/journal.pone.0025148)
Supplement: Table S1 — Example scenarios. Three examples are provided for each of the five types of moral dilemmas viewed by study participants. (DOCX) [file pone.0025148.s001.docx]

## Table S1

| **Scenario type** | **Example** |
| --- | --- |
| *Intended harm* | *Example 1:* “Eric is hiking along a cliff when a rockslide occurs. A boulder is rolling toward five people on the trail and the cliffs are too steep for escape. A sixth hiker is sitting in a tree over the cliff. By pushing hard on the tree, Eric could knock this hiker out of the tree and into the boulder’s path, stopping it and saving the five. Eric can push on the tree and the one hiker will die; or he can refrain from doing this, letting the five die.”  *Example 2:* “Adam is standing near a drawbridge overlooking train tracks and sees a train approaching. Five people are trapped by steep banks on the tracks below. Near Adam is a switch that will raise the bridge, dropping a man on the bridge onto the tracks and stopping the train. Adam can throw the switch, killing the one man, or he can refrain from doing this, letting the five die.”  *Example 3:* “Faith is leading a tour over a high bridge. A section of the bridge starts to give way. Five people wind up trapped on a section that is about to collapse, and one person is safely standing near a large plank. Faith can push on this plank and knock the person across the gap and the five can cross safely over his body, but the person will be killed by the impact. Faith can push on the plank and the one person will die; or she can refrain from doing this, letting the five die.” |
| *Foreseen harm* | *Example 1:* “Eric is hiking along a cliff when a rockslide occurs. A boulder is rolling toward five people on the trail and the cliffs are too steep for escape. By pushing hard on a tree near the trail Eric could knock it in the boulder’s path and force the boulder down the cliff, saving the five. There is a hiker on a trail directly below who would be killed, though. Eric can push on the tree and the one hiker will die; or he can refrain from doing this, letting the five die.  *Example 2:* “Adam is standing near a bridge overlooking train tracks and sees a train approaching. Five people are trapped by steep banks on the tracks below. Near Adam is a switch that will divert the train onto a different track where one person is trapped. Adam can throw the switch, killing the one man; or he can refrain from doing this, letting the five die.”  *Example 3:* “Faith is leading a tour over a high bridge. A section of the bridge starts to give way. Five people wind up trapped on a section that is about to collapse, and one person is clinging to a nearby plank. Faith can push on this plank so that it reaches the five trapped people and allows them to cross to safety, but the person clinging to it will fall and be killed. Faith can push the plank and the one person will die; or she can refrain from doing this, letting the five die.” |
| *Neutral* | *Example 1:* “Eric is hiking along a cliff when a rockslide occurs. A boulder is rolling down the trail that Eric wants to hike down. By pushing hard on a tree near the trail, Eric could knock it in the boulder’s path so the boulder will not block his trail. Eric can push on the tree and the trail will remain clear; or he can refrain from doing this, and the trail will be blocked.  *Example 2*: “Adam is standing near a bridge overlooking train tracks and sees a train approaching. If it continues on the same track, the train will go to Springfield, the wrong destination. Near Adam is a switch that will divert the train onto a different track towards Fairview, the correct destination. Adam can throw the switch and the train will go to Fairview; or he can refrain from doing this, letting the train go to Springfield.”  *Example 3:* “Faith is leading a tour over a high bridge. A plank in the middle of the bridge tells the history of the bridge. Faith can push on the plank so it is out of sight, and the tourists will not be able to read it. Faith can push the plank and the tourists will not be able to read it; or she can refrain from doing this and the tourists will be able to read it.” |
| *No gain* | *Example 1:* “Eric is hiking along a cliff when a rockslide occurs. A boulder is rolling down a trail. A person is sitting in the tree alongside the trail. By pushing hard on the tree, Eric could knock this person out of the tree and into the boulder’s path. Eric can push on the tree and the person will die; or he can refrain from doing this, and no one die.”  Example 2: “Adam is standing near a bridge overlooking train tracks and sees a train approaching on the track that runs below the bridge. Near Adam is a switch that will divert the train onto a different track where a person is trapped. Adam can throw the switch, killing the man; or he can refrain from doing this and no one will die.”  *Example 3:* “Faith is leading a tour over a high bridge. A section of the bridge starts to give way. One person winds up safely standing near a large plank. Faith can push on this plank so that the person falls through the gap, and the person will be killed. Faith can push the plank and the person will die; or she can refrain from doing this, and no one will die. |
| *No cost* | *Example 1:* “Eric is hiking along a cliff when a rockslide occurs. A boulder is rolling toward five people on the trail and the cliffs are too steep for escape. By pushing hard on a tree near the trail Eric could knock it in the boulder’s path and force the boulder down the cliff, saving the five. Eric can push on the tree and no one will die; or he can refrain from doing this, letting the five die.”  *Example 2:* “Adam is standing near a bridge overlooking train tracks and sees a train approaching. Five people are trapped by steep banks on the tracks below. Near Adam is a switch that will divert the train onto a different track where no people are trapped. Adam can throw the switch and no one will die; or he can refrain from doing this, letting the five die.”  *Example 3:* “Faith is leading a tour over a high bridge. A section of the bridge starts to give way. Five people wind up trapped on a section that is about to collapse. Faith can push on a nearby plank so that it reaches the five trapped people and allows them to cross to safety. Faith can push on the plank and no one will die; or she can refrain from doing this, letting the five die.” |
